# Influence of Molecular Weight on Conversion and *exo*-Selectivity in Thermal PIBSA Synthesis from Highly Reactive Polyisobutylene

**DOI:** 10.3390/polym18182197

**Published:** 2026-09-09

**Authors:** Syafiqa Mohd. Saleh, Ahmad Shalabi Md. Sauri, Mohd Fazli Mohammat, Fatin Nur Amira Mohammad Hejemi, Mifzal Mohamad Nazrel, Karimah Kassim, Ahmad Zafir Romli, Putri Nur Arina Mohd Ariff, Ahmad Faiza Mohd, Noor Hidayah Pungot, Aimi Suhaily Saaidin, Agustono Wibowo, Mohamad Faizzudin Mat Piah, Jamali Basar

**Affiliations:** 1 PETRONAS Research Sdn. Bhd, Lot 3288,3289, Off Jalan Ayer Itam, Kawasan Institusi Bangi, Kajang 43000, Selangor, Malaysia; syafiqa_saleh@petronas.com (S.M.S.); shalabi.sauri@petronas.com (A.S.M.S.); faizzudin.matpiah@petronas.com (M.F.M.P.); jamali_basar@petronas.com (J.B.); 2 Organic Synthesis Laboratory, Institute of Science, Universiti Teknologi MARA (UiTM), Puncak Alam Campus, Puncak Alam, Kuala Selangor 42300, Selangor, Malaysia; fatinnuramira1234@gmail.com (F.N.A.M.H.); mifzalnazrel@gmail.com (M.M.N.); karimah@uitm.edu.my (K.K.); putriarina17@gmail.com (P.N.A.M.A.); noorhidayah977@uitm.edu.my (N.H.P.); aimisuhaily@uitm.edu.my (A.S.S.); agustono@uitm.edu.my (A.W.); 3 Polymer Characterization Research Laboratory, Institute of Science, Universiti Teknologi MARA (UiTM), Puncak Alam Campus, Puncak Alam, Kuala Selangor 42300, Selangor, Malaysia; ahmad349@uitm.edu.my (A.Z.R.); ahmadfaiza@uitm.edu.my (A.F.M.)

**Keywords:** HR-PIB, PIBSA, lubricants, dispersants, full factorial

## Abstract

Polyisobutylene succinic anhydride (PIBSA) is an important intermediate for ashless dispersant additives used in lubricant formulations, where conversion and *exo*-selectivity strongly influence product performance. In this work, the thermal synthesis of PIBSA derived from highly reactive polyisobutylene (HR-PIB) 1300 and 2300 was systematically investigated using a full factorial experimental design to evaluate the effects of feed molar ratio, reaction temperature, and stirring rate on conversion and *exo*-PIBSA yield. Structural confirmation was performed using ^1^H NMR spectroscopy, gel permeation chromatography (GPC), and saponification value analysis. HR-PIB 1300 achieved conversions of 60–94%, while HR-PIB 2300 achieved conversions of 75–88% under the investigated conditions. A clear molecular-weight-dependent trade-off between conversion and *exo*-selectivity was observed. For PIBSA 1300, higher temperatures increased conversion but reduced selectivity, resulting in *exo*-PIBSA yields of 24–56%. In contrast, PIBSA 2300 achieved yields of up to 71%, highlighting the importance of mixing and mass-transfer effects in higher-viscosity systems. These findings demonstrate that optimal thermal maleation conditions are strongly dependent on polymer molecular weight and provide mechanistic insight for future optimization of industrial PIBSA production.

## 1. Introduction

Polyisobutylene succinic anhydride (PIBSA) is one of the most important intermediates used in the production of ashless dispersant additives for lubricant and fuel formulations. PIBSA-derived dispersants play a critical role in preventing sludge formation, soot agglomeration, and varnish deposition in internal combustion engines, thereby improving engine cleanliness, thermal stability, and lubricant lifetime [1,2]. Increasing global demand for high-performance lubricants, together with stricter environmental and fuel-efficiency regulations, has accelerated interest in more efficient and sustainable PIBSA production technologies [2].

**Scheme 1:**
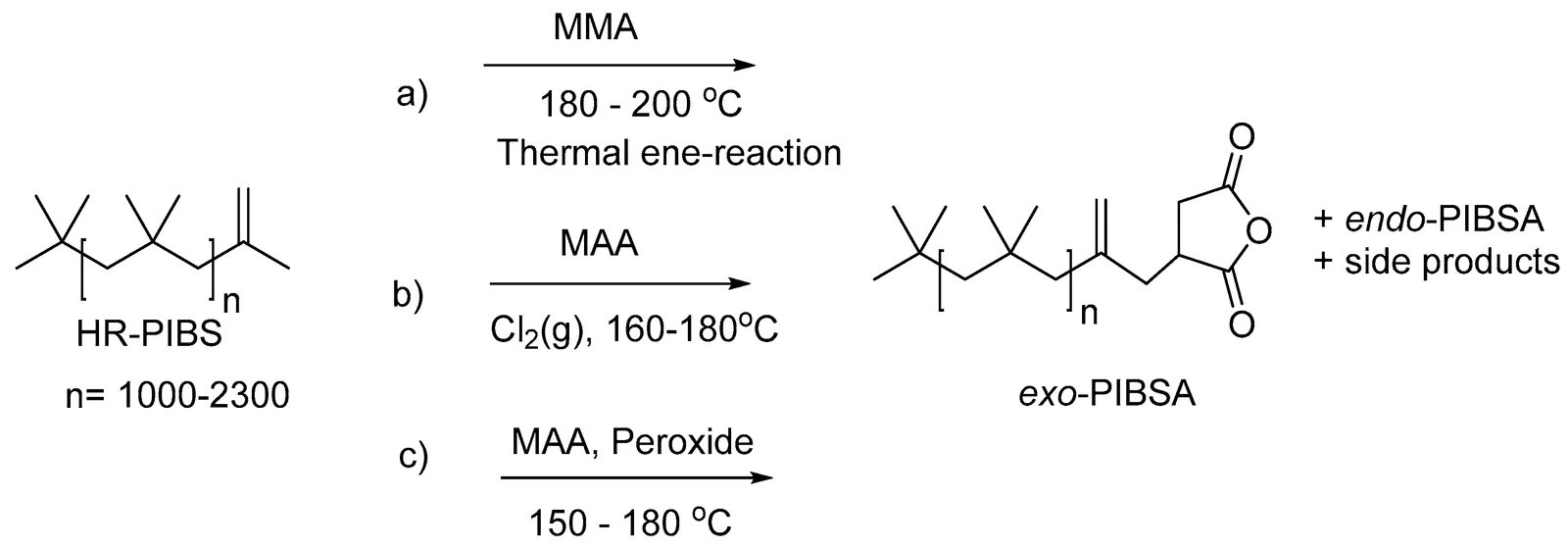
Synthesis of PIBSA from HR-PIB and maleic anhydride (MAA).

Industrial PIBSA synthesis is commonly achieved through the thermal maleation of highly reactive polyisobutylene (HR-PIB) with maleic anhydride via an Alder-ene reaction [3]. Compared with chlorination-assisted processes or radical initiated peroxide reaction, thermal synthesis is increasingly preferred because it eliminates residual chlorine or radical species contamination and reduces environmental and handling concerns associated with chlorinated intermediates [3,4]. However, the thermal ene process requires relatively severe operating conditions, typically involving temperatures above 180 °C and extended reaction times, which often promote competing side reactions, thermal degradation, and reduced product selectivity [5,6]. The general reaction scheme for PIBSA formation from HRPIB and maleic anhydride is shown in Scheme 1.

**Figure 1:**
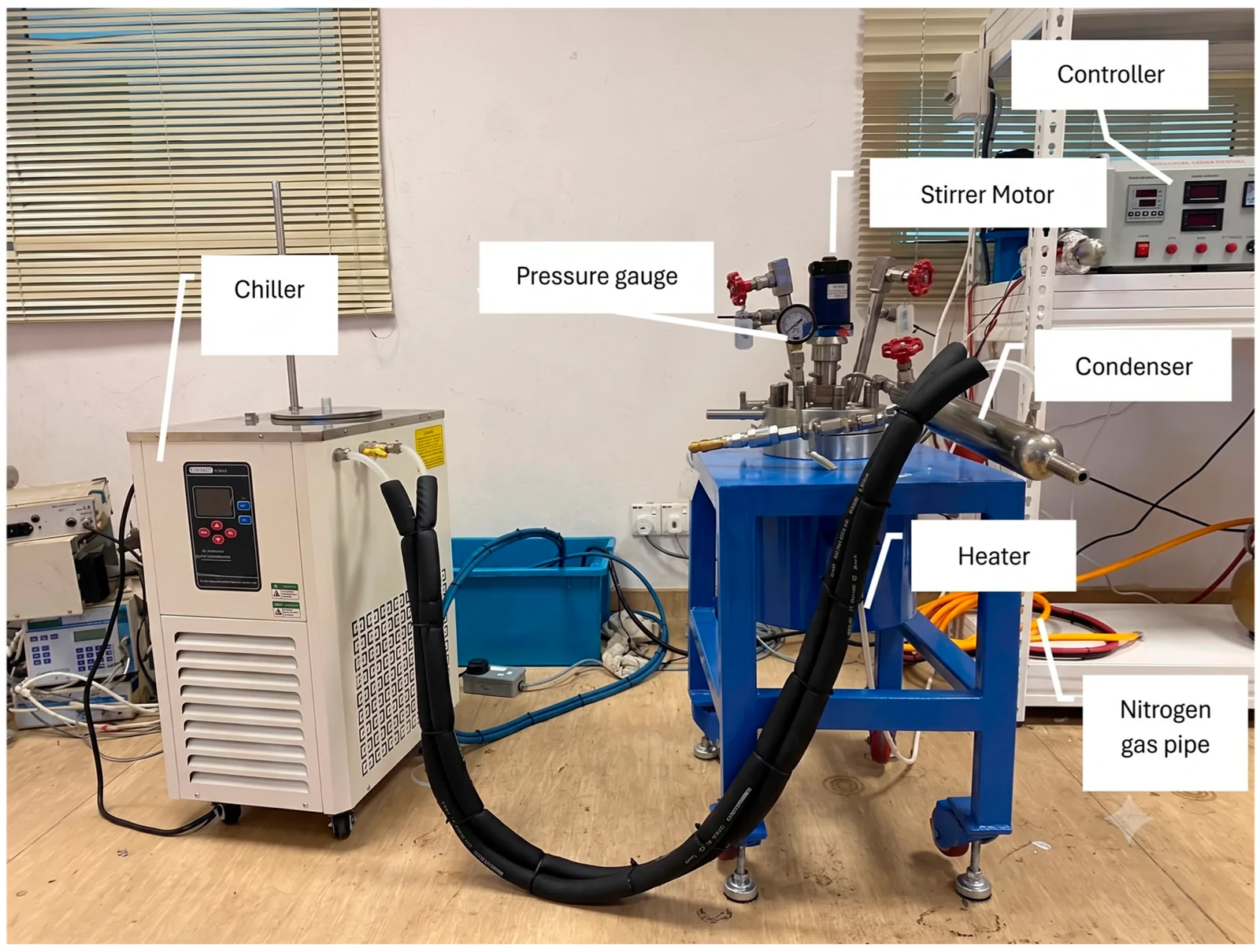
2L Stainless steel reactor setup.

Despite the widespread industrial application of PIBSA, controlling the balance between conversion and *exo*-selectivity remains a major challenge. Previous mechanistic investigations demonstrated that elevated temperatures accelerate maleation reactions but simultaneously promote secondary pathways, including isomerization, over-maleation, and formation of undesired by-products [7]. These competing reactions reduce the yield of *exo*-PIBSA, which is generally regarded as the preferred product due to its higher reactivity toward subsequent dispersant synthesis [8]. Recent kinetic studies further revealed that the thermal maleation process is highly sensitive to reaction environment, oxygen content, and mixing conditions, emphasizing the complex interplay between reaction kinetics and transport phenomena in concentrated polymer systems [3]. The general reactor setup for thermal maleation is as shown in Figure 1.

Most published studies on PIBSA synthesis have focused primarily on low molecular weight PIB systems, typically within the Mn range of 500–1000 g mol^−1^, where viscosity effects remain relatively manageable [9]. In contrast, higher molecular weight PIB systems remain considerably less explored despite their importance in heavy-duty lubricants, marine engines, and oilfield applications [10]. Increasing polymer molecular weight significantly alters reaction behavior by increasing viscosity, reducing molecular mobility, and limiting mass transfer efficiency within the reaction medium. These effects can strongly influence reactant accessibility, heat transfer, and local concentration profiles, ultimately affecting conversion, selectivity, and product distribution during thermal maleation [11,12].

Furthermore, although statistical optimization approaches such as factorial experimental design and response surface methodology have been widely applied in chemical process development [13,14], systematic investigations correlating molecular weight effects with thermal PIB maleation behavior under reactor-scale conditions remain limited. In particular, comparative understanding of how process parameters influence the conversion–selectivity relationship for PIBSA synthesized from different HR-PIB molecular weights has not been comprehensively reported.

Therefore, this work investigates the thermal synthesis of PIBSA derived from HR-PIB 1300 and HR-PIB 2300 using a full factorial experimental design to evaluate the effects of feed molar ratio, reaction temperature, and stirring rate on conversion and *exo*-PIBSA yield. Structural characterization was performed using ^1^H NMR spectroscopy, gel permeation chromatography (GPC), and saponification value analysis. Particular emphasis was placed on understanding the molecular-weight-dependent trade-off between conversion and selectivity, as well as the influence of mixing and transport limitations under reactor-scale thermal ene-reaction conditions. The findings provide mechanistic insight into PIBSA process behavior and establish a basis for future optimization of industrial dispersant precursor synthesis.

## 2. Materials and Methods

### 2.1. Materials

HR-PIB M1300 and M2300 were obtained from the in-house production facility of Petronas, Kuala Lumpur, Malaysia. Maleic anhydride (99%) (Sigma Aldrich), Tetrahydrofuran, 99.8% (stabilized with 0.025% BHT) (Merck), Dichloromethane (99.5%) (Merck) were purchased from Systerm Malaysia Sdn Bhd (Shah Alam, Malaysia). All these materials were used as received, without further purification.

#### 2.1.1. General Procedure for Synthesis of PIBSA 1300 and 2300 at Laboratory Scale

Highly reactive polyisobutylene, HR-PIB 1300 (10.0 g, 7.69 mmol), was charged into a nitrogen-purged reaction vessel equipped with a Liebig condenser and magnetic stirring. Maleic anhydride was then added at two molar ratios (2.25 equivalents) (17.3 mmol) and 3.0 equivalents (23.1 mmol), relative to HR-PIB 1300. As for HR-PIB 2300 (10.0 g, 4.35 mmol), it was reacted with maleic anhydride at 3.0 equivalents (13.0 mmol) and 4.0 equivalents (17.4 mmol), relative to HR-PIB 2300. Prior to heating, each reaction mixture was purged with nitrogen for 30 min to minimize oxygen- and moisture-induced side reactions. The reaction mixtures were heated at 200–215 °C for 5–10 h under continuous stirring. After completion, the crude products were cooled to room temperature and subjected to vacuum treatment to remove residual maleic anhydride, resulting in honey-colored PIBSA products. Product formation was confirmed by ^1^H NMR spectroscopy using CDCl_3_ as the deuterated solvent. The disappearance or reduction in olefinic HR-PIB signals and the appearance of characteristic PIBSA resonances were used to confirm successful maleation. The successful grafting process was further supported by gel permeation chromatography (GPC) analysis, where an increase in the average molecular mass relative to the parent HR-PIB 1300 and HR-PIB 2300 was observed following maleic anhydride incorporation.

#### 2.1.2. General Procedure for Synthesis of PIBSA 1300 and 2300 at Reactor Scale

**Figure 2:**
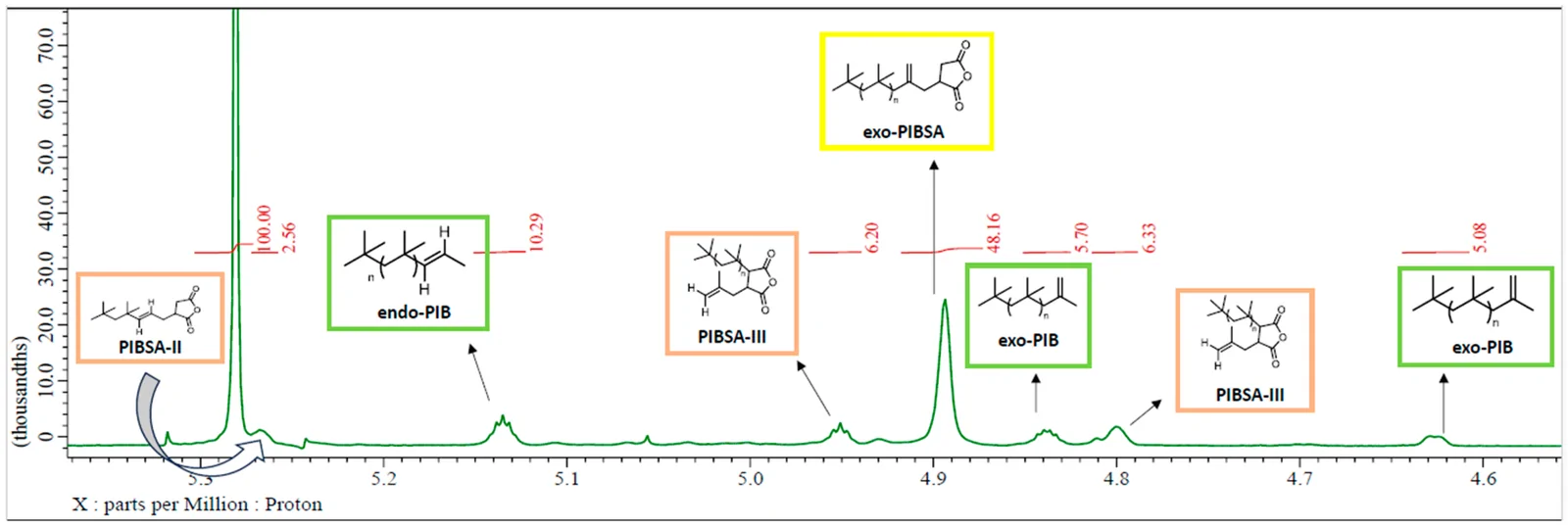
^1^H NMR spectrum of PIBSA 1300 synthesized at laboratory scale.

HR-PIB Grade 1300 (500 g, 0.38 mol) and HR-PIB Grade 2300 (500 g, 0.22 mol) were separately reacted with maleic anhydride at varying molar equivalents in a 2 L stainless steel reactor (Figure 2). For HR-PIB 1300, maleic anhydride was added at two equivalents (0.77 mol) and three equivalents (1.15 mol), while HR-PIB 2300 was reacted at three equivalents (0.65 mol) and four equivalents (0.87 mol), relative to the respective HR-PIB feed.

All reactions were conducted under a pressurized system at 3 bar to maintain the reaction medium in the liquid phase and minimize vaporization during the thermal maleation process. Prior to the reaction, the empty stainless-steel reactor was purged with nitrogen gas to remove oxygen and moisture that could promote unwanted side reactions or oxidative degradation. Subsequently, 500 g of highly reactive polyisobutylene (HR-PIB) was introduced into the reactor, and heating was initiated gradually to avoid overshooting the target reaction temperature. A second nitrogen purge was then carried out for 5 min to ensure an inert reaction environment and maintain product stability throughout the reaction.

Maleic anhydride (MAA) was preheated separately until fully molten before being introduced into the reactor containing HR-PIB. The reaction mixtures were maintained under continuous stirring for 5 h under the specified reaction conditions. The reaction parameters investigated using a full factorial design of experiments (DOE) are summarized in Table 1 and Table 2 for PIBSA 1300 and PIBSA 2300, respectively. The evaluated parameters included feed molar ratio (2–3 equivalents for PIBSA 1300 and 3–4 equivalents for PIBSA 2300), reaction temperature (200–220 °C), and stirring rate (150–400 rpm).

The upper and lower limits of these operating ranges were established based on preliminary laboratory-scale reactions conducted at the 10 g scale. Separate screening experiments were performed for HR-PIB 1300 and HR-PIB 2300 at 500 g to identify practical operating windows prior to reactor-scale evaluation and to minimize unnecessary material consumption. Consequently, the two HR-PIB grades were not investigated over an identical factorial domain.

The conversion of HR-PIB and the yield of *exo*-PIBSA were determined using ^1^H NMR spectroscopy. The conversion, selectivity, and yield were calculated using the following equations:

$$Conversion\;\left(\%\right)=\;\left(\frac{HR\;PIB\;Reacted}{HR\;PIB\;Initially\;fed}\right)\times \;\;100\%$$

$$Selectivity\;\left(\%\right)=\left(\frac{Exo-PIBSA\;produced}{HR\;PIB\;Reacted}\right)\times \;100\%$$

$$Yield\;\left(\%\right)=\left(\frac{Exo-PIBSA\;produced}{HR\;PIB\;Initially\;fed}\right)\times \;100\%$$

The experimental data were statistically analyzed using analysis of variance (ANOVA) with Design-Expert software (Version 23.1.0, Stat-Ease Inc., Minneapolis, MN, USA). The experimental responses were fitted to the most appropriate statistical model based on the significance of the model terms and goodness-of-fit parameters.

### 2.2. Characterization

#### 2.2.1. GPC Analysis

Gel permeation chromatography (GPC) analyses were performed using a Shimadzu (Kyoto, Japan) LC-20A chromatographic system equipped with a refractive index detector (RID-10A), an autosampler, a pump, and an injection loop of 100 μL. The system was fitted with three columns (300 mm × 8 mm) connected in series (GPC-803, GPC-804, and GPC-805). Tetrahydrofuran (THF) was used as both the solvent and mobile phase. Analyses were conducted at 40 °C with a flow rate of 1.0 mL min^−1^ using polymer solutions prepared at a concentration of 0.5 wt%. Polystyrene standards with mass-average molar masses ranging from 2500 to 1,355,000 g mol^−1^ and dispersity values close to unity were used for calibration and determination of average molar masses.

#### 2.2.2. Saponification Number

The saponification number of the samples was determined according to ASTM D94 [15]. Approximately 2.0 g of sample was accurately weighed into a round-bottom flask, followed by the addition of 25 mL of toluene to ensure complete dissolution. A blank determination was prepared using the same procedure without the sample.

Subsequently, 25 mL of ethanolic potassium hydroxide (KOH) solution was added to both the sample and blank solutions. The mixtures were refluxed at 80 °C for 2 h to ensure complete saponification. After completion of the reaction, the solutions were cooled to room temperature, and 2 mL of phenolphthalein indicator was added. The excess KOH was then titrated with 0.5 M hydrochloric acid (HCl) until the endpoint was reached. Initial and final burette readings were recorded for both the blank and sample titrations. The saponification number was calculated using the standard saponification value equation based on the difference in titrant volume between the blank and sample solutions.

#### 2.2.3. Nuclear Magnetic Resonance Spectroscopy Analysis

^1^H NMR spectra were acquired using a JEOL (Tokyo, Japan) 400 MHz spectrometer with dichloromethane (DCM, 99.5%) employed as the internal standard. For reactor-scale samples, 10 g of product was collected, and DCM was added at a 1:1 molar ratio relative to HR-PIB to enable quantitative analysis. The mixture was stirred mechanically for 2 min until complete dissolution was achieved.

Subsequently, approximately 60 mg of the resulting solution was dissolved in 1 mL of deuterated chloroform (CDCl_3_) and transferred into an NMR tube for analysis. The integrals of the characteristic NMR signals corresponding to *exo*-PIBSA, *endo*-PIBSA (PIBSA-II), PIBSA-III, *endo*-PIB, and unreacted *exo*-PIB were measured relative to the DCM reference peak for determination of conversion, selectivity, and product distribution.

## 3. Results

### 3.1. Synthesis at 10 g Laboratory Scale

PIBSA grades 1300 and 2300 were initially synthesized at the laboratory scale to establish a preliminary understanding of the thermal maleation behavior of highly reactive polyisobutylene (HR-PIB). The transformation proceeds through a thermally induced Alder-ene reaction between HR-PIB and maleic anhydride, typically occurring at temperatures above 150 °C. Under these conditions, multiple reaction products may be generated, including *exo*-PIBSA, *endo*-PIBSA (PIBSA-II), and secondary by-products collectively referred to as PIBSA-III (Figure 2) and other by-products. The formation of these species is strongly influenced by the thermal reaction environment and the accessibility of allylic hydrogen atoms along the PIB backbone.

**Scheme 2:**
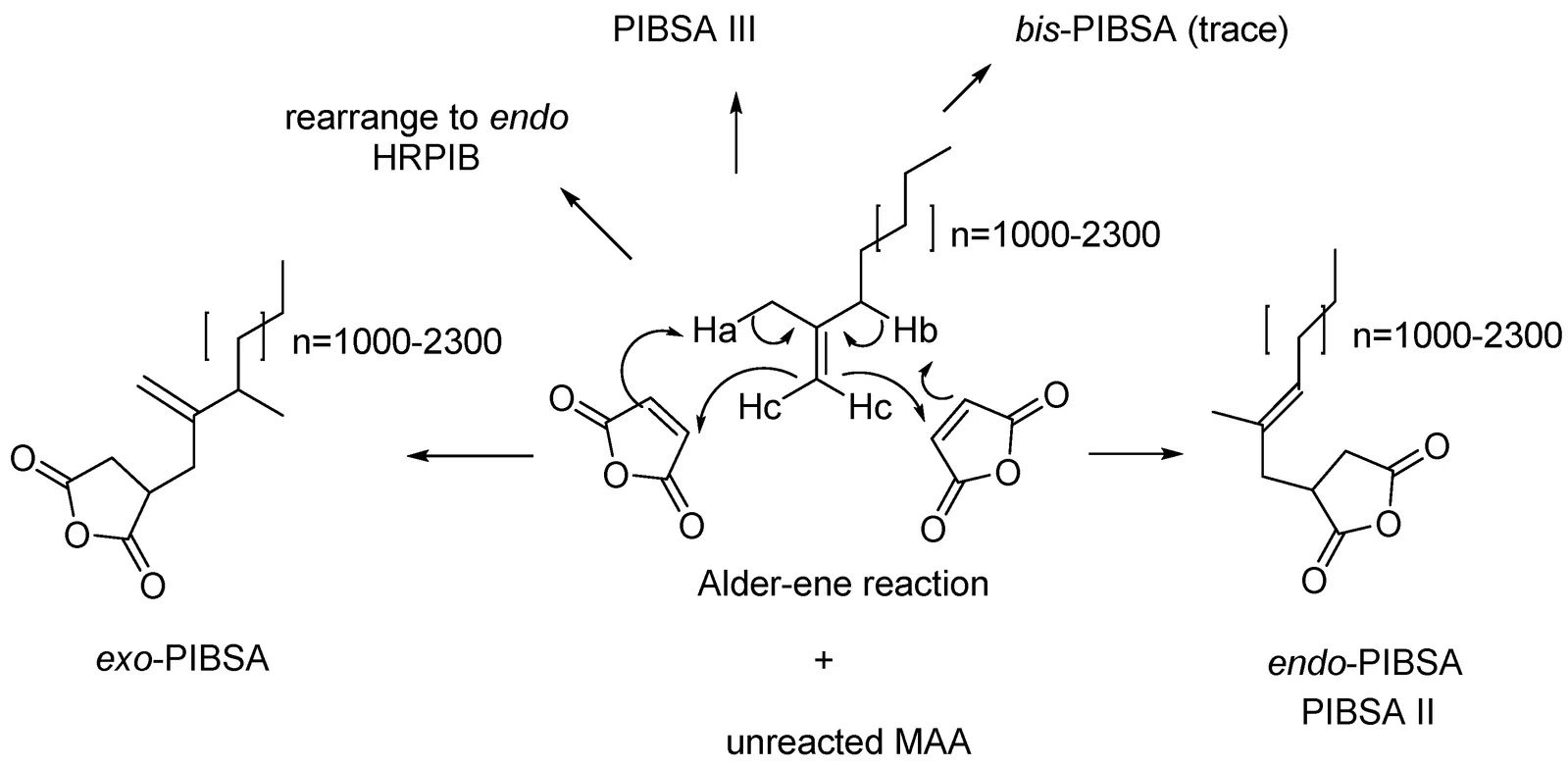
Mechanistic reaction for PIBSA products under thermal ene reaction.

**Figure 3:**
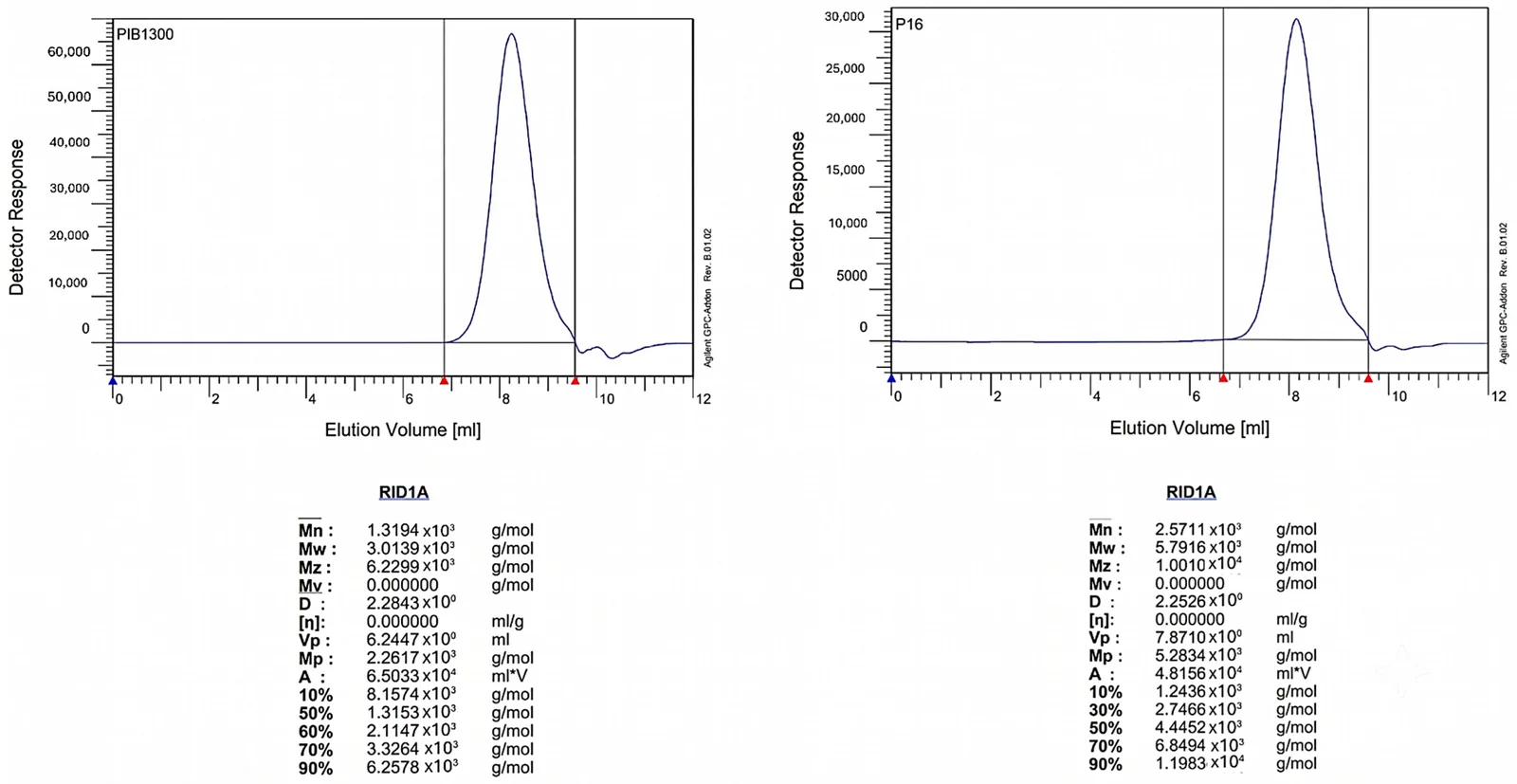
GPC Analyses of PIBSA 1300 and PIBSA 2300.

Under thermal ene-reaction conditions, *exo*-PIBSA was identified as the predominant product. This behavior is consistent with the preferred allylic hydrogen transfer pathway involving terminal *exo*-olefinic sites of HR-PIB, where lower steric hindrance favors formation of the *exo*-adduct. In contrast, formation of endo-PIBSA (PIBSA-II) and PIBSA-III is associated with secondary thermal rearrangement pathways and competing side reactions occurring under elevated reaction temperatures. The proposed reaction mechanism for thermal PIB maleation is illustrated in Scheme 2. Residual unreacted *exo*-PIB and its rearranged *endo*-PIB isomer were observed in the spectra of both PIBSA 1300 and PIBSA 2300, indicating the coexistence of incomplete conversion and thermally induced olefin isomerization during the maleation process (Figure 2 and Figure 3). Similar product distributions and thermal rearrangement behavior have previously been reported for PIB maleation systems [16].

Analysis of the ^1^H NMR spectra revealed a substantial reduction in the characteristic olefinic proton signals at δ = 4.6–4.9 ppm, confirming significant consumption of terminal double bonds during the grafting reaction. Simultaneously, the characteristic resonance corresponding to maleic anhydride at δ = 7.0 ppm was either absent or significantly diminished in the final products, indicating effective incorporation of maleic anhydride into the PIB backbone rather than the presence of residual unreacted monomer. These spectral changes collectively confirm successful thermal grafting of succinic anhydride functionalities onto the HR-PIB chains.

This observation is further supported by gel permeation chromatography (GPC) analysis, which revealed an increase in molecular weight following the thermal maleation reaction. The observed shift toward higher molecular mass is consistent with successful grafting of succinic anhydride functionalities onto the HR-PIB backbone (Figure 3: Mn = 1319 and Mn = 2571 g/mol). Together with the NMR and saponification value results (Table 3), the GPC data provide complementary evidence for successful PIBSA formation. Comparable NMR-based characterization approaches have also been employed in related PIBSA and PIBSI systems to evaluate grafting efficiency and product composition [8].

**Figure 4:**
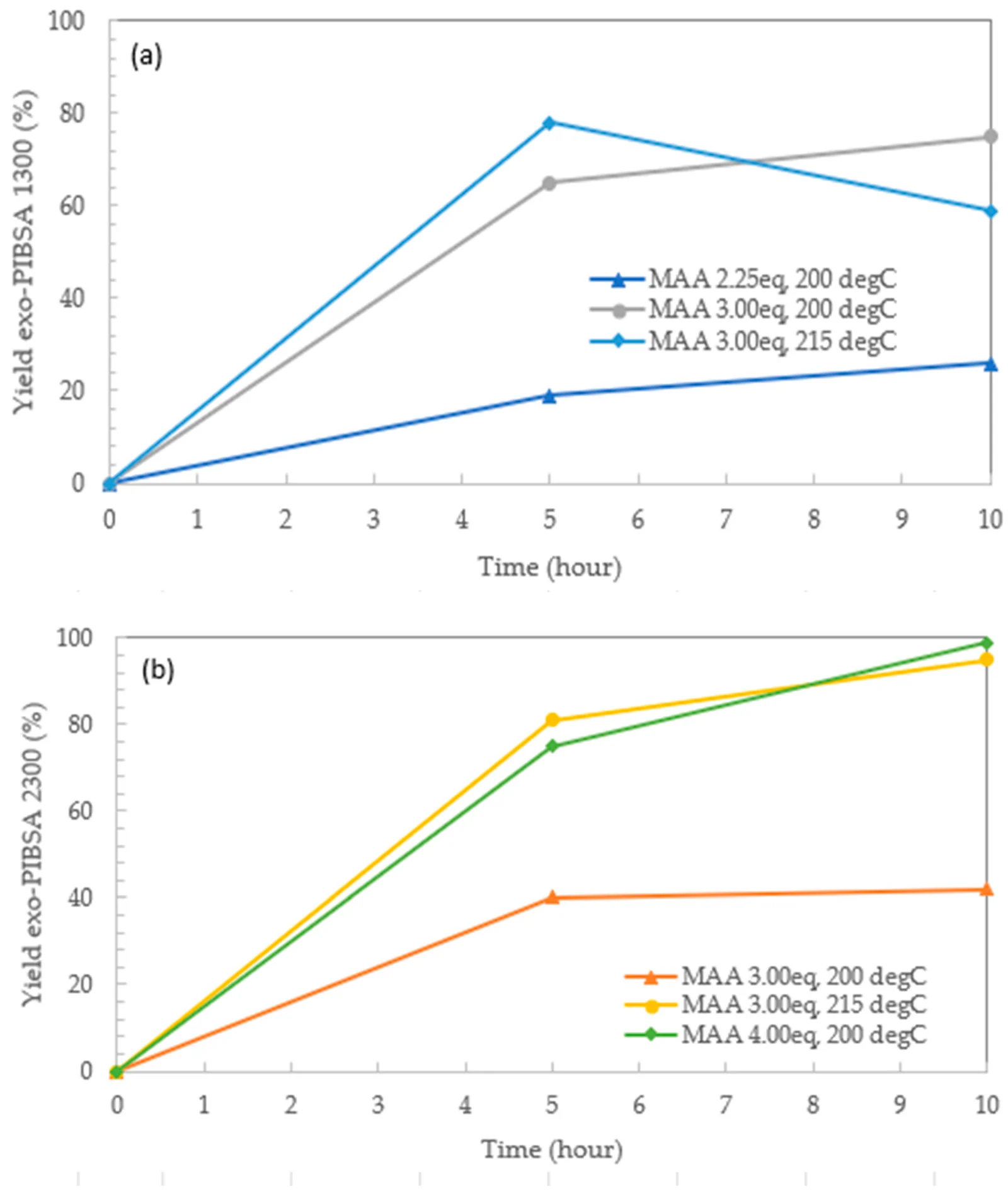
(**a**) Yield of PIBSA 1300 synthesized at 10 g laboratory scale; (**b**) PIBSA 2300 synthesized at 10 g laboratory scale.

Figure 4 compares the progress of *exo*-PIBSA yield over time for PIBSA 1300 and PIBSA 2300 (Figure 3), synthesized at laboratory scale under different reaction conditions. For all conditions, the yield increases significantly within the first 5 h of reaction with slight improvement in the next 5 h of operation. The analysis is thus focused on the 5 h reaction time to reflect a more industrially relevant condition, as longer reaction durations would increase operational costs (OPEX) without necessarily significantly improving yield.

For PIBSA 1300 (Figure 4a), the highest initial yield is observed at 3.0 equivalents MAA at 215 °C, reaching 78% at 5 h. Increasing the MAA loading from 2.25 to 3.0 equivalents at 200 °C significantly enhances the yield, highlighting the importance of sufficient reactant availability for reaction. Furthermore, increasing the reaction temperature from 200 °C to 215 °C at 3.0 equivalents leads to an additional increase in yield, indicating that higher thermal energy accelerates the ene reaction and improves conversion. For PIBSA 2300 (Figure 4b), a similar trend is observed at the 5 h reaction time, where increasing both the maleic anhydride loading and reaction temperature leads to a significant improvement in *exo*-PIBSA yield.

While the trends are well established at the 10 g scale, direct scale-up to 500 g without systematic evaluation may introduce risks such as heat transfer limitations, mass transfer inefficiencies, and mixing non-uniformity, all of which can impact yield and reproducibility. Following structural confirmation, the reactor-scale synthesis of PIBSA 1300 and PIBSA 2300 were subjected to factorial analysis to systematically evaluate process sensitivity. The experimental runs are detailed as in Table 1 and Table 2.

### 3.2. Production of PIBSA 1300 at 500 g Scale

The results obtained from the reaction of HR-PIB 1300 with maleic anhydride are summarized in Table 3. PIBSA synthesized under varying thermal maleation conditions exhibited substantial differences in both saponification value and visual appearance, reflecting differences in the degree of functionalization and extent of secondary thermal reactions. Samples with saponification values within the range of 80–110 mg KOH g^−1^ consistently exhibited lighter coloration, suggesting controlled incorporation of succinic anhydride functionalities onto the polyisobutylene backbone with relatively limited side reactions. In contrast, samples exhibiting saponification values exceeding 130 mg KOH g^−1^ appeared significantly darker, indicating increased thermal severity and possible over-functionalization during the maleation process.

The darker appearance of these samples is likely associated with excessive incorporation of succinic anhydride groups, which promotes competing secondary reactions such as oxidative degradation, thermal condensation, oligomerization, and possible intermolecular crosslinking. Under elevated thermal conditions, these side reactions may generate tar and chromophoric by-products, resulting in progressive darkening of the reaction mixture. Similar observations have previously been reported in thermally induced PIB maleation systems and highly reactive polyisobutylene synthesis, where prolonged exposure to elevated temperatures increased the formation of thermally degraded and oxidized species [7]. Despite the occurrence of these competing pathways, the conversion of HR-PIB 1300 remained consistently high across the operating window investigated, ranging from 60% to 94% (Table 3).

ANOVA and diagnostic analysis of the initial full-factorial dataset identified Runs 5 and 7 as highly influential observations, exhibiting externally studentized residuals exceeding conventional screening thresholds and elevated Cook’s distance values. No analytical or procedural error could be conclusively identified for these experiments. Since the objective of the factorial design is as preliminary factor screening rather than process optimization, the reduced dataset is used to explore potential first-order trends in conversion behavior.

Statistical evaluation using analysis of variance (ANOVA) demonstrated that the developed model was statistically significant, with a model *p*-value of 0.0496 at the 95% confidence level (Table 4). Furthermore, the model exhibited high coefficient of determination (R^2^ = 0.9667), suggesting that the selected process variables adequately described the conversion behavior within the investigated experimental domain.

The high conversion observed for HR-PIB 1300 suggests that thermal *ene*-reaction kinetics were sufficiently promoted under the applied reaction conditions. However, the simultaneous increase in saponification value and formation of darker products under more severe conditions indicates that maximizing conversion alone does not necessarily correspond to improved process selectivity. This observation highlights the inherent competition between conversion and selective *exo*-PIBSA formation during thermal maleation, particularly under conditions favoring excessive thermal activation.

**Figure 5:**
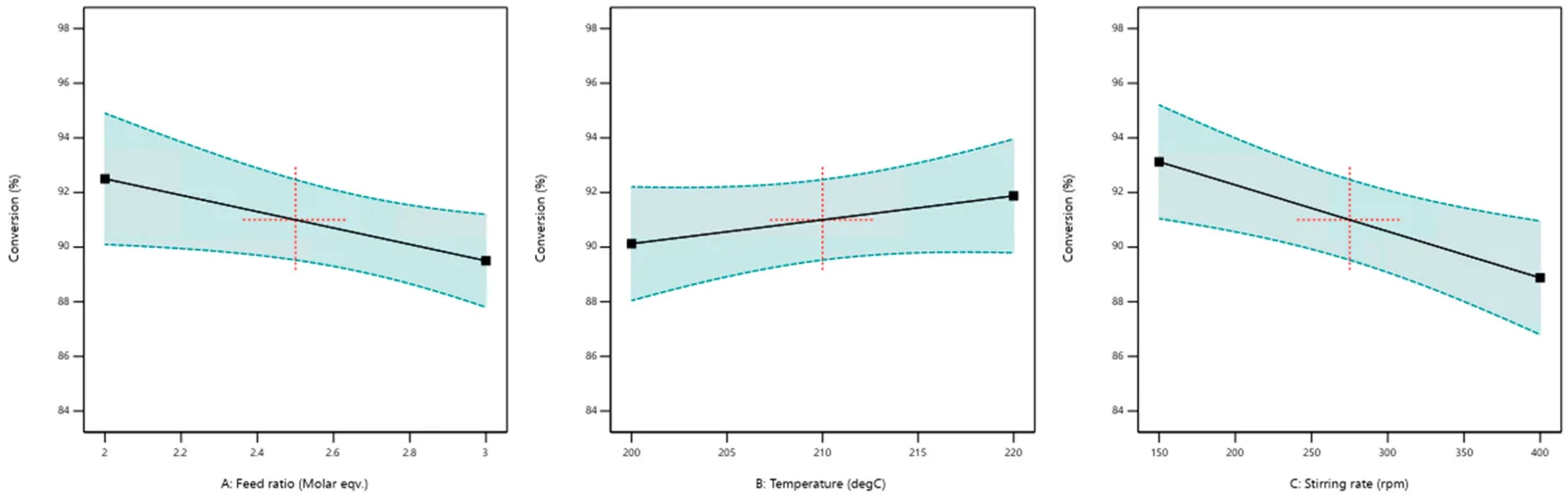
(**A**) Feed ratio for HR-PIB 1300 conversion; (**B**) Temperature for HR-PIB 1300 conversion; (**C**) Stirring rate for HR-PIB 1300 conversion.

Statistical analysis identified stirring rate and feed molar ratio as the dominant parameters influencing the conversion of HR-PIB 1300, followed by reaction temperature. Based on the ANOVA results, the relative significance of the investigated variables can be ranked as: stirring rate > feed ratio > temperature. The factors profiler shown in Figure 5 indicate that higher conversion was generally achieved at lower stirring rates, lower feed ratios, and elevated reaction temperatures within the investigated operating window.

The strong sensitivity of conversion toward stirring rate highlights the important role of fluid-dynamics and mass-transfer behavior during thermal maleation of HR-PIB systems. Although mixing is essential for maintaining homogeneous reactant distribution in viscous polymer media, excessive agitation may also influence local thermal distribution and reactant residence behavior, potentially promoting competing side reactions and reducing the effective utilization of maleic anhydride for productive grafting. In relatively lower viscosity HR-PIB 1300 systems, the thermal ene reaction appears to remain predominantly kinetically controlled; therefore, elevated temperatures strongly enhance reaction kinetics and increase overall conversion by promoting allylic hydrogen transfer and maleic anhydride incorporation into the polymer backbone.

The influence of feed ratio further suggests that excessive maleic anhydride loading does not necessarily improve conversion efficiency under thermal conditions. At higher feed concentrations, secondary maleation pathways and competing thermal reactions may become increasingly significant, leading to reduced selectivity and less efficient utilization of the reactive olefinic sites. Similar transport and reaction-environment effects have previously been reported in viscous polymer modification systems, where molecular mobility and local concentration gradients strongly influence grafting efficiency and product distribution [12,17].

Despite the consistently high conversion observed for HR-PIB 1300, achieving high selectivity toward *exo*-PIBSA remained considerably more challenging. The selectivity ranged from 27% to 73%, corresponding to moderate *exo*-PIBSA yields between 24% and 56%.

Statistical analysis of the screening dataset suggested that reaction temperature, feed ratio, and stirring rate may influence *exo*-PIBSA yield within the investigated operating window. The reduced first-order model provided an initial framework for evaluating the relative direction and magnitude of factor effects, indicating the trend: temperature > feed ratio > stirring rate (Table 5). The model is significant, with *p*-value of 0.0153 and a good coefficient of determination (R^2^ = 0.9564). The developed model for *exo*-PIBSA yield was found in contrast to conversion behavior. However, because the present work employed an unreplicated full-factorial design, the resulting model should be interpreted primarily as an exploratory screening tool for identifying potentially important process variables and assessing the suitability of the selected factor ranges rather than as a predictive optimization model.

**Figure 6:**
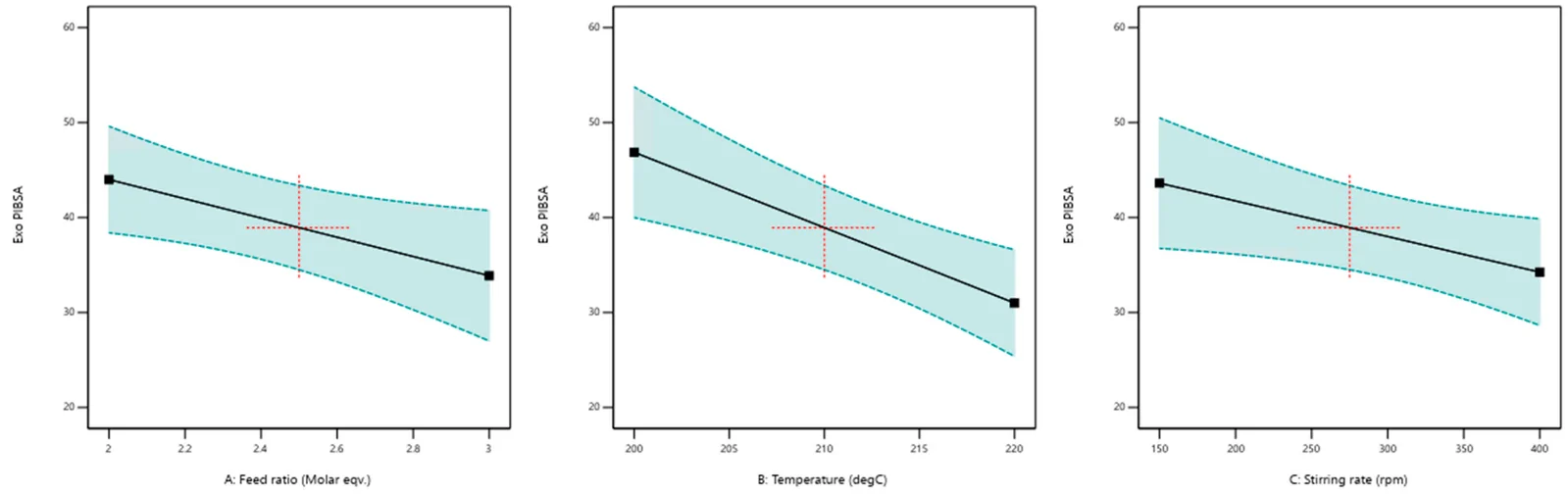
(**A**) Feed ratio for the yield of *exo*-PIBSA 1300; (**B**) Temperature for the yield of *exo*-PIBSA 1300; (**C**) Stirring rate for the yield of *exo*-PIBSA 1300.

The factors profiler presented in Figure 6 demonstrates that lower reaction temperatures, reduced feed ratios, and lower stirring rates favor higher *exo*-PIBSA yields. This behavior highlights the inherent competition between reaction conversion and selective *exo*-adduct formation during thermal maleation. At elevated temperatures, the Alder-ene-reaction proceeds more rapidly due to increase in the thermodynamic effects [18]. However, the higher thermal energy simultaneously promotes secondary pathways, including olefin isomerization, over-maleation, thermal rearrangement, and formation of PIBSA-II and PIBSA-III species [19]. Consequently, although conversion increases under more severe conditions, selective formation of *exo*-PIBSA becomes progressively less favorable.

The strong dependence of *exo*-selectivity on temperature indicates that selective PIBSA formation is governed not only by reaction kinetics but also by the balance between productive ene-reaction pathways and competing thermally induced side reactions. These findings demonstrate that maximizing conversion alone is insufficient for increasing PIBSA synthesis, as excessive thermal severity compromises product selectivity and overall *exo*-PIBSA yield.

Based on statistical analysis, reaction temperature exerted the strongest influence on *exo*-PIBSA yield, followed by feed molar ratio and stirring rate. This behavior highlights the dominant role of thermal energy in governing the balance between productive Alder-ene-reaction pathways and competing side reactions during PIB maleation.

A pronounced trade-off between conversion and *exo*-selectivity was observed as a function of reaction temperature. Lower reaction temperatures favored higher *exo*-PIBSA selectivity despite moderately reduced conversion. Under these conditions, the ene-reaction pathway appears to proceed in a more controlled manner, where steric and kinetic factors favor selective allylic hydrogen transfer from terminal *exo*-olefinic sites of HR-PIB. Consequently, formation of the desired *exo*-adduct is enhanced while competing thermal rearrangement and secondary maleation pathways remain relatively suppressed [5].

In contrast, increasing the reaction temperature to 200–220 °C significantly accelerated the overall maleation process, leading to higher conversion of HR-PIB. However, the elevated thermal energy simultaneously promoted competing side reactions, including olefin isomerization, over-maleation, and formation of PIBSA-II and PIBSA-III species. These thermally induced pathways progressively reduced *exo*-selectivity and limited the overall *exo*-PIBSA yield despite the higher extent of conversion. Similar mechanistic behavior has previously been reported in thermal PIB maleation systems, where excessive thermal activation shifted the reaction environment toward less selective product distributions [6].

**Figure 7:**
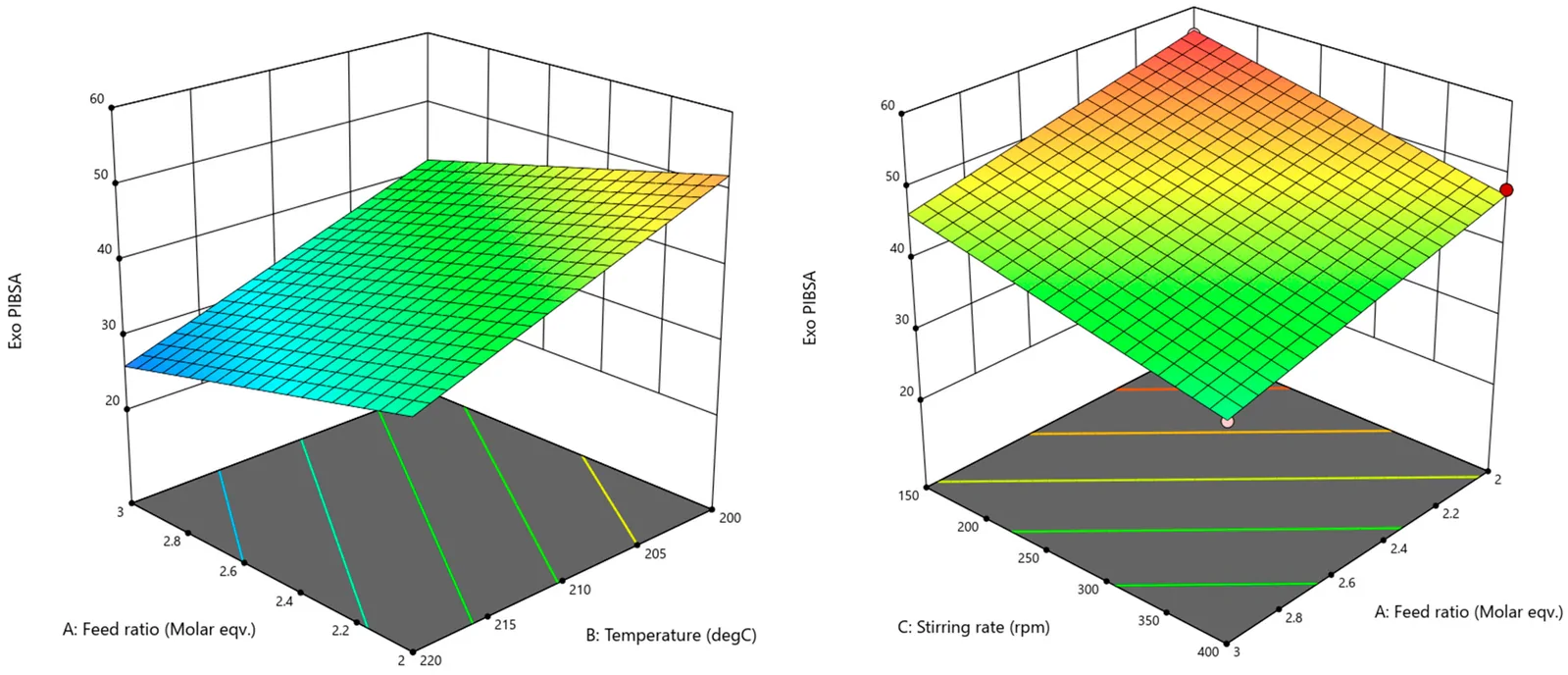
Model graph 3D surface for yield of *exo*-PIBSA 1300.

The three-dimensional response surface shown in Figure 7 further illustrates the complex interaction between reaction temperature and feed molar ratio on *exo*-PIBSA yield. Within the investigated experimental domain, the maximum *exo*-PIBSA yield was limited to approximately 56%, indicating that the current operating conditions remain insufficient to fully suppress competing side reactions under thermal maleation conditions. The response surface suggests that the reaction system approaches a selectivity-limited regime at elevated temperatures and higher maleic anhydride loadings, where additional thermal activation no longer translates into improved productive grafting efficiency.

Previous studies have demonstrated that lower reaction temperatures in the range of 150–180 °C may significantly improve *exo*-selectivity by reducing secondary thermal pathways [3]. Within the investigated operating range, the screening model suggests that lower feed ratios and reduced thermal severity may be beneficial for *exo*-PIBSA formation. These conditions warrant further investigation in future optimization studies.

It should be noted that the response surface and optimization trends presented here are derived from the 500 g system and therefore reflect scale-dependent behavior. As observed earlier, these trends differ from those obtained at the 10 g laboratory scale, where higher MAA loading and elevated temperatures led to significantly higher yields. At larger scale, however, the influence of mixing efficiency, heat transfer, and mass transport limitations becomes more pronounced, leading to reduced yields and altered parameter sensitivities. These scale-related effects may introduce concentration and temperature gradients within the reactor, thereby impacting selectivity and overall conversion. Consequently, the identified optimum should be interpreted within the context of the 500 g system, highlighting the importance of reactor design and operating conditions in governing process performance upon scale-up.

### 3.3. Production of PIBSA 2300 at 500 g Scale

The products obtained from thermal maleation of HR-PIB 2300 are summarized in Table 6. Similar to PIBSA 1300, the conversion of HR-PIB 2300 remained consistently high across the investigated operating window, ranging from 75% to 88%, indicating that the applied thermal conditions were sufficient to achieve substantial olefin conversion. In contrast to PIBSA 1300, however, PIBSA 2300 exhibited systematically lower saponification values, which can be attributed primarily to its significantly higher molecular weight. The longer polymer chains reduce the relative concentration of succinic anhydride functionalities per unit mass, resulting in lower measured saponification values despite successful grafting.

During the preliminary model development, diagnostic analysis identified Run 2 as a highly influential observation, exhibiting a substantially larger externally studentized residual and Cook’s distance relative to the remaining experiments. Because the present full-factorial design was intended as a screening study to identify potentially important variables and assess the direction of factor effects, a reduced dataset is used, and first-order model was examined to explore underlying process trends. The resulting model is interpreted as an exploratory screening model rather than a predictive optimization model.

**Figure 8:**
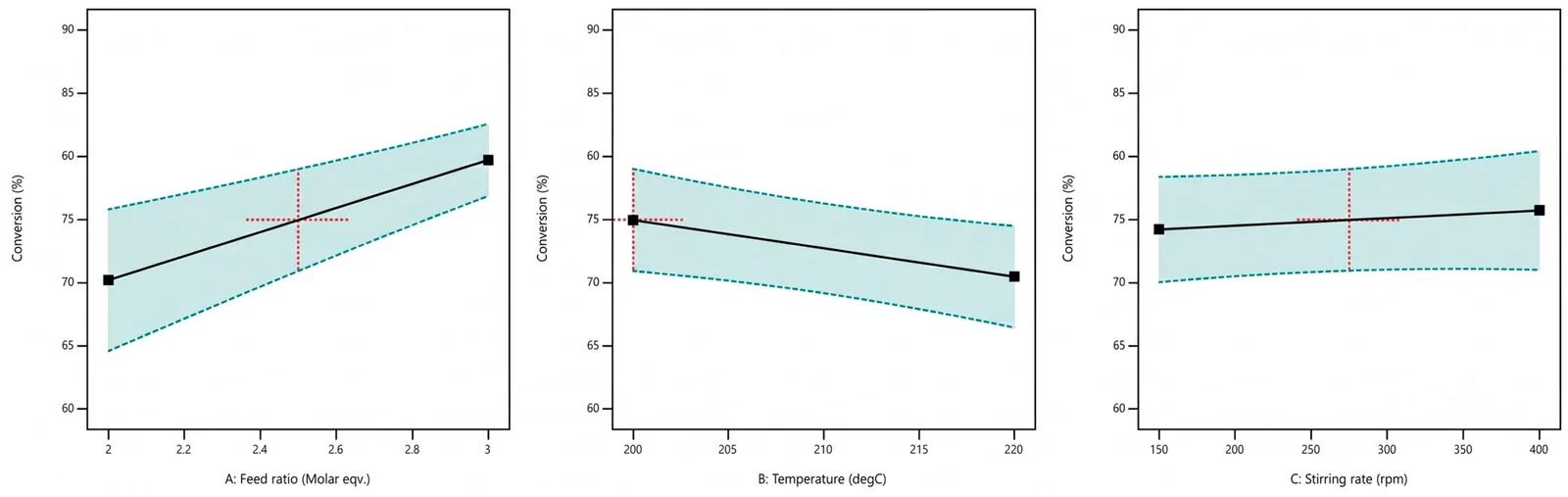
(**A**) Feed ratio for HR-PIB 2300 conversion; (**B**) Temperature for HR-PIB 2300 conversion; (**C**) Stirring rate for HR-PIB 2300 conversion.

The ANOVA results for HR-PIB 2300 conversion (Table 7) indicate that the overall model is statistically significant (*p* = 0.0200). The relative significance of the investigated variables can be ranked as: feed ratio > temperature > stirring rate. In contrast to HR-PIB 1300, where stirring rate was the dominant factor, this shift indicates that HR-PIB 2300 is less responsive to mixing within the studied range and more strongly governed by reactant stoichiometry. This behavior is likely due to its higher molecular weight and viscosity, which imposes greater mass transfer limitations and reduces the effectiveness of the applied stirring conditions. As inferred from the factors profiler in Figure 8, it is plausible that at a wider MAA loadings range (e.g., 3–5 equivalents) and enhanced mixing intensity may be required to shift the system out of the diffusion-limited regime and reveal stronger process sensitivities for HR-PIB 2300 conversion.

Despite the high conversion levels, *exo*-PIBSA yields for PIBSA 2300 ranged from 36% to 71%. However, the corresponding first-order model was not statistically significant (*p* = 0.2564), indicating that the investigated factors were insufficient to explain the observed yield variability within the studied operating window. Therefore, no statistically supported conclusions regarding the influence of feed ratio, temperature, or stirring rate on *exo*-PIBSA 2300 yield can be drawn from the present dataset (Table 8).

This suggested that the reaction behavior of higher molecular weight HR-PIB systems is governed by more complex interactions beyond the investigated operating parameters alone. It indicates that additional factors such as viscosity-induced transport limitations, localized concentration gradients, and reactor fluid dynamics may exert increasingly important effects on product distribution in higher molecular weight systems.

In addition to differences in conversion and selectivity, variations in the physical appearance of the PIBSA 2300 products were also observed under different reaction conditions. Samples synthesized under higher feed ratios and lower stirring efficiencies generally exhibited darker coloration together with elevated saponification values. This behavior is likely associated with increased local thermal severity and over-functionalization during the maleation process. Under such conditions, it is hypothesized that excessive incorporation of succinic anhydride functionalities may promote secondary reactions including thermal oxidation, oligomerization, and formation of tar-like species, which contribute to progressive darkening of the product [20]. However, no direct characterization was conducted in the present study to confirm these mechanisms. Similar observation was in agreement with PIBSA 1300 before.

Nevertheless, the extent of discoloration observed for PIBSA 2300 was generally less pronounced than that observed for PIBSA 1300 (except Table 6, entry (1) due to formation excessive tar-like products). This behavior may be attributed to the significantly higher molecular weight of HR-PIB 2300, where the larger polymer backbone effectively lowers the relative concentration of succinic anhydride functionalities per unit mass. Consequently, the probability of excessive local over-maleation and thermally induced side-product formation may be partially reduced compared with lower molecular weight PIB systems. The comparatively lower saponification values observed for PIBSA 2300 further support this interpretation, indicating a lower overall grafting density despite successful maleation.

The higher viscosity associated with HR-PIB 2300 likely reduces molecular mobility and restricts effective mass transfer within the reaction medium. Under such conditions, localized concentration heterogeneity and diffusion limitations become increasingly significant, limiting effective reactant accessibility and reducing selective ene-reaction efficiency. Consequently, higher stirring rates become necessary to improve mixing efficiency, reduce concentration gradients, and enhance contact between maleic anhydride and the reactive olefinic sites along the polymer backbone. Simultaneously, lower feed ratios help suppress excessive local maleic anhydride concentrations that may otherwise promote secondary thermal pathways and over-functionalization.

These findings demonstrate that the governing factors controlling thermal PIB maleation are strongly dependent on polymer molecular weight and reaction environment. While PIBSA 1300 synthesis remains predominantly influenced by reaction kinetics and thermal activation, PIBSA 2300 synthesis becomes increasingly sensitive to fluid-dynamic and transport-related effects arising from increased polymer viscosity. Similar transport limitations and mixing-dependent behavior have previously been reported in highly viscous polymer processing systems [11,12].

**Figure 9:**
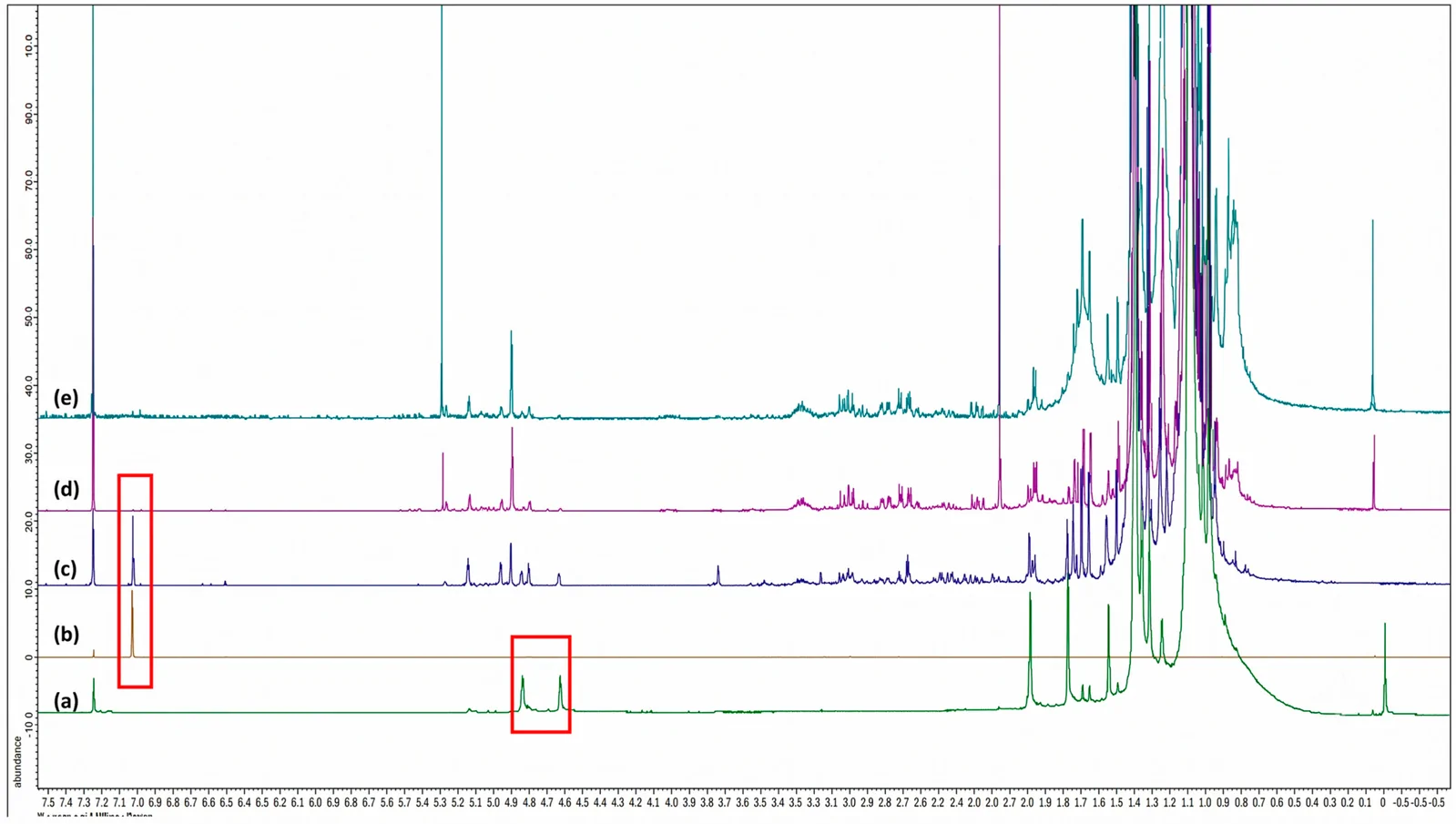
^1^H NMR of the materials: (**a**) HR-PIB 1300; (**b**) Maleic Anhydride; (**c**) PIBSA 1300 Lab Scale; (**d**) PIBSA 1300 Reactor Scale; (**e**) PIBSA 2300 Reactor Scale.

Encouragingly, the reproducibility of the spectral features across both laboratory-scale and reactor-scale PIBSA 1300 and PIBSA 2300 samples (Figure 9), demonstrates the successful scale-up of the thermal maleation process without significant alteration of the overall product distribution. The chromatograms did not exhibit substantial peak broadening or the appearance of high-molecular-weight shoulders, suggesting that extensive intermolecular crosslinking or polymer degradation did not occur under the investigated reaction conditions. The relatively narrow molecular-weight distributions indicate that maleation predominantly proceeded through functionalization of the existing polymer chains rather than significant alteration of the polymer architecture.

## 4. Conclusions

The thermal synthesis of PIBSA derived from HR-PIB 1300 and HR-PIB 2300 was systematically investigated under reactor-scale conditions using a full factorial experimental design to evaluate the effects of feed molar ratio, reaction temperature, and stirring rate on conversion and *exo*-selectivity. Both HR-PIB systems achieved consistently high conversion under the investigated operating conditions; however, a clear molecular-weight-dependent trade-off between conversion and *exo*-selectivity was observed.

For PIBSA 1300, higher reaction temperatures promoted conversion but reduced *exo*-selectivity due to increased side reactions and competing thermal pathways. In contrast, although high conversion was achieved for PIBSA 2300, no statistically significant model was obtained for *exo*-PIBSA yield. Consequently, the effects of the process investigated variables on yield could not be established with confidence within the studied experimental domain. These findings demonstrate that the dominant process parameters governing PIBSA synthesis vary significantly according to HR-PIB molecular weight, and therefore optimal thermal maleation conditions cannot be generalized across different PIB grades. Further investigation is required to confirm the underlying mechanisms.

The present study provides preliminary insight that polymer molecular weight exerts a significant influence on the thermal maleation behavior of highly reactive polyisobutylene. While differences in response between HR-PIB 1300 and HR-PIB 2300 were observed, additional experimental evidence is required to confirm the mechanistic role of viscosity and transport-related effects. The observed conversion–selectivity trade-off highlights the importance of balancing thermal activation and process severity to maximize *exo*-PIBSA formation.

Future work should incorporate replicated experiments and center points to establish experimental variability and improve statistical confidence in the observed trends. Response surface methodology (RSM) will subsequently be employed to evaluate curvature effects, quantify factor interactions, and develop robust optimization models for reactor-scale PIBSA synthesis. Additional characterization, including viscosity measurements and transport-related analyses, could help to verify the mechanistic interpretations proposed in this study.

Collectively, these findings provide an initial framework for understanding molecular-weight-dependent behavior during thermal PIB maleation and establish a basis for more comprehensive optimization studies aimed at industrial PIBSA production.

## Figures and Tables

**Table 1:** Full factorial for PIBSA 1300 production.

Run	Feed Ratio (Molar eq)	Temperature (°C)	Stirring Rate (rpm)
1	3	220	150
2	3	220	400
3	3	200	400
4	3	200	150
5	2	220	150
6	2	220	400
7	2	200	400
8	2	200	150

**Table 2:** Full factorial for PIBSA 2300 production.

Run	Feed Ratio (Molar eq)	Temperature (°C)	Stirring Rate (rpm)
1	4	220	150
2	4	220	400
3	4	200	400
4	4	200	150
5	3	220	150
6	3	220	400
7	3	200	400
8	3	200	150

**Table 3:** PIBSA 1300 results.

Run	Feed Ratio	Temp. (°C)	Stirring Rate	Conversion * (%)	Selectivity ** (%)	Yield *** (%)	Saponification Value	Appearance
1	3	220	150	93	30	28	137.77	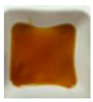
2	3	220	400	88	27	24	114.02	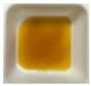
3	3	200	400	87	43	37	110.51	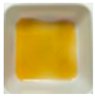
4	3	200	150	90	29	26	131.91	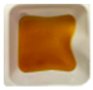
5	2	220	150	60	73	44	84.30	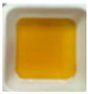
6	2	220	400	91	31	28	134.66	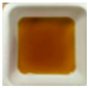
7	2	200	400	78	62	48	85.42	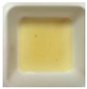
8	2	200	150	94	60	56	85.35	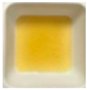

All parameters (*,**,***) were determined using ^1^H NMR.

**Table 4:** ANOVA result for HR-PIB 1300 conversion.

Source	Sum of Squares	df	Mean Square	F-Value	*p*-Value	
**Model**	36.25	3	12.08	19.33	0.0496	significant
A-Feed ratio	12.00	1	12.00	19.20	0.0483	
B-Temperature	4.08	1	4.08	6.53	0.1250	
C-Stirring rate	24.08	1	24.08	38.53	0.0250	
**Residual**	1.25	2	0.6250			
**Cor Total**	37.50	5				

**Table 5:** ANOVA result for yield of *exo*-PIBSA 1300.

Source	Sum of Squares	df	Mean Square	F-Value	*p*-Value	
**Model**	819.48	3	273.16	21.93	0.0153	significant
A-Feed ratio	164.03	1	164.03	13.17	0.0360	
B-Temperature	403.23	1	403.225	32.37	0.0108	
C-Stirring rate	140.63	1	140.625	11.29	0.0437	
**Residual**	37.38	3	12.46			
**Cor Total**	856.86	6				

**Table 6:** PIBSA 2300 results.

Run	Feed Ratio	Temp. (°C)	Stirring Rate	Conversion * (%)	Selectivity ** (%)	Yield *** (%)	Saponification Value	Appearance
1	4	220	400	86	51	44	87.63	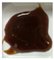
2	4	200	400	77	82	63	107.91	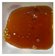
3	4	200	150	88	41	36	118.12	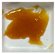
4	3	220	150	75	77	58	82.30	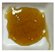
5	3	220	400	75	95	71	90.07	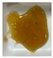
6	3	200	400	81	53	43	72.90	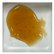
7	3	200	150	79	67	53	84.13	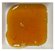

All parameters (*,**,***) were determined using ^1^H NMR.

**Table 7:** ANOVA result for HR-PIB 2300 conversion.

Source	Sum of Squares	df	Mean Square	F-Value	*p*-Value	
**Model**	147.33	3	49.11	49.11	0.0200	significant
A-Feed ratio	120.33	1	120.33	120.33	0.0082	
B-Temperature	27.00	1	27.00	27.00	0.0351	
C-Stirring rate	3.00	1	3.00	3.00	0.2254	
**Residual**	2.00	2	1.00			
**Cor Total**	149.33	5				

**Table 8:** ANOVA result for yield of *exo*-PIBSA 2300.

Source	Sum of Squares	df	Mean Square	F-Value	*p*-Value	
**Model**	665.50	3	221.83	3.05	0.2564	not significant
A-Feed ratio	484.00	1	484.00	6.66	0.1230	
B-Temperature	49.00	1	49.00	0.6743	0.4979	
C-Stirring rate	400.00	1	400.00	5.50	0.1436	
**Residual**	145.33	2	72.67			
**Cor Total**	810.83	5				

## Data Availability

The original contributions presented in this study are included in the article. Further inquiries can be directed to the corresponding author.

## Abbreviations

The following abbreviations are used in this manuscript:

HR-PIB	Highly Reactive Polyisobutene)
MAA	Maleic anhydride
PIBSA	Polyisobutylene Succinic Anhydride
PIBSI	Polyisobutylene Succinimide
Mn	Number-Average Molecular Weight
GPC	Gel Permeation Chromatography
DOE	Design of experiments
RSM	Response Surface Methodology
